# A novel approach to treatment of priapism refractory to non-surgical methods: A single-case experience

**DOI:** 10.1080/2090598X.2019.1626130

**Published:** 2019-06-12

**Authors:** Nassib Abou Heidar, Jad A. Degheili, Gerges Bustros, Wassim Wazzan, Muhammad Bulbul

**Affiliations:** Department of Surgery, Division of Urology, American University of Beirut Medical Center, Beirut, Lebanon

**Keywords:** Priapism, cavernosal infusion, phenylephrine

## Abstract

Ischaemic priapism is a rarely encountered urological emergency that can lead to erectile dysfunction if not treated. Treatment strategies for ischaemic priapism include: cavernosal sympathomimetic injections; percutaneous shunts; and surgical procedures including shunts and penile prostheses. We present a case of a middle-aged man presenting with ischaemic priapism refractory to cavernosal injections and percutaneous T-shunt procedure. After refusal of surgery, a continuous cavernosal infusion of phenylephrine was successfully performed with resolution of erection and no sequelae. For the treatment of ischaemic priapism, adherence to management guidelines should be encouraged; however, it is still a disease entity that is not well understood and new treatment protocols may have a role in the future.

**Abbreviation**: ED: erectile dysfunction

## Introduction

Ischaemic priapism is a urological emergency resulting in complete erectile dysfunction (ED) if treatment is delayed [1]. Treatment strategies for ischaemic priapism, as in most medical conditions, gradually increases in invasiveness from local injections to invasive shunt procedures, and eventually surgical shunting procedures and recently an increasing trend towards early penile implant insertion [2]. We hereby present a case of ischaemic priapism refractory to cavernosal injections and percutaneous transglandular shunts who was eventually successfully treated by continuous intracavernosal infusion of phenylephrine.

## Case presentation

**Figure 1.**
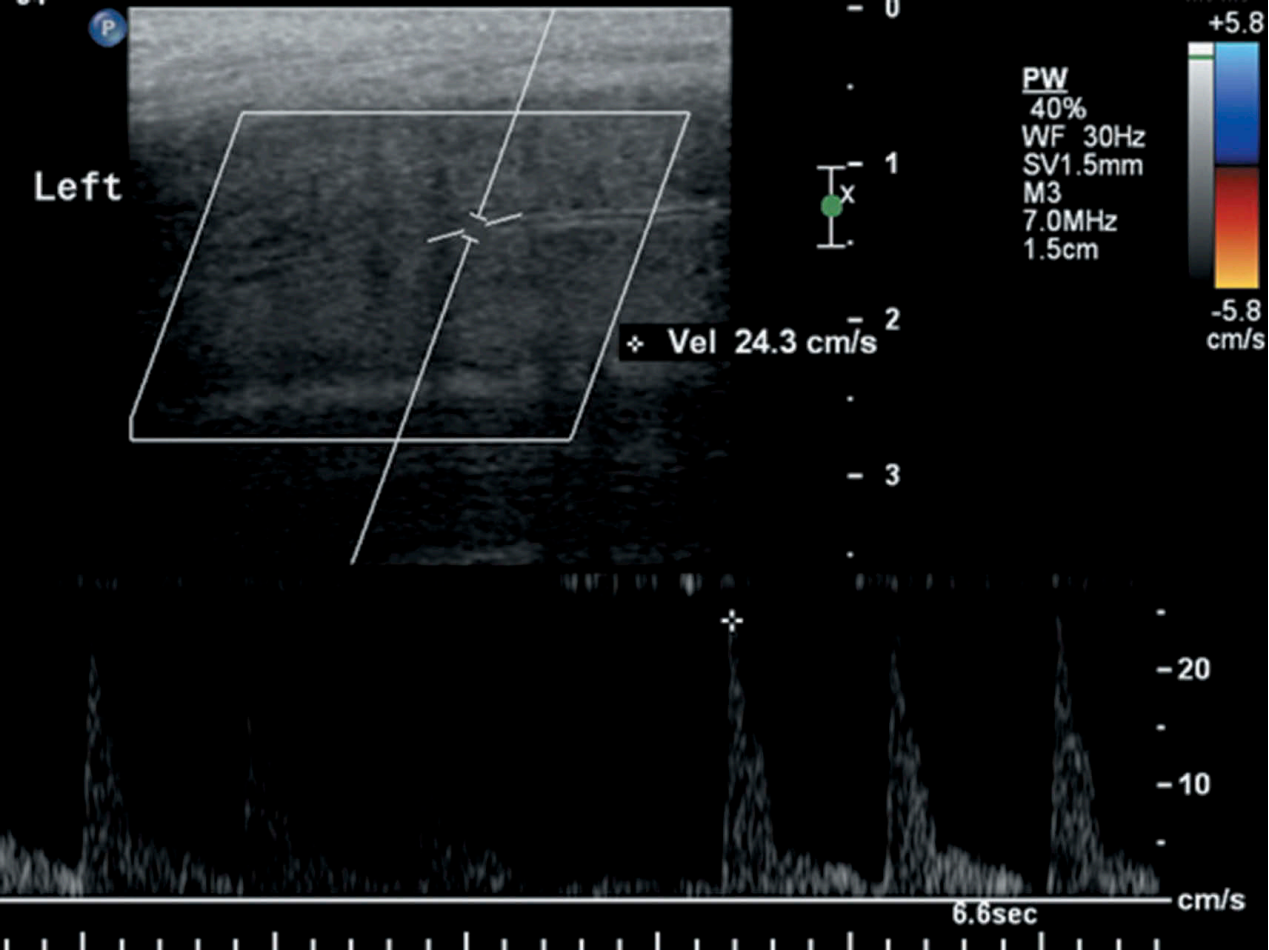
Ultrasound Doppler of the penile artery showing adequate flow after continuous phenylephrine infusion.

A 58-year-old man presented to the Emergency Department with a 24-h history of continuous painful erection after ingestion of a 20 mg tadalafil pill, without prescription. Upon examination, the penis was rigid and severely tender, no skin discoloration was noted. Initial management started by inserting an i.v. line with 10 L/min oxygen using a facemask. Evacuation of all blood clots was then performed after performing a penile block, using an 18-G angiocatheter inserted through the glans to the corpora cavernosa bilaterally until fresh blood was obtained, followed by copious irrigation with normal saline. The erection did not subside. We then preceded with intracavernosal injection of phenylephrine in aliquots of 200 µg every 3–5 min, reaching a maximum dose of 1 mg every hour. A partial response was achieved, but soon after, the patient would have a recurrent painful erection. The same serial intracavernosal injection was repeated several times with a partial response and recurrent painful erection each time. A T-shunt was done with minimal response. The patient was advised to undergo a surgical shunt; however, he was very adamant about not undergoing surgery. We decided to admit the patient to a monitored bed with a cavernosal 21-G butterfly needle followed by continuous infusion of phenylephrine at a rate of 1 mg/h for 8 h continuously overnight, with monitoring of vital signs and cardiac rhythm. The patient was consented that this method is not standard of care and experimental, and that there is a risk of infection with such treatment. To avoid infection, the needle site was heavily cleaned with betadine and serial examinations of the site were performed. After accomplishing this protocol, erection consequently subsided and an ultrasound Doppler of the penis was performed showing good bilateral penile arterial flow with a peak systolic velocity of 26 cm/s and an end diastolic velocity of 7.2 cm/s, which are in the normal range, with no significant fibrosis of the corpora or any calcified plaques (Figure 1) and there were no sequelae at the needle site. The patient subsequently regained his baseline erections on follow-up and was advised not to use tadalafil or any other medications without medical counselling.

## Discussion

The AUA defines priapism as a continuous erection that is unrelated to, or persists after sexual stimulation, for >4 h. Its incidence encompasses 0.3–1 case per 100 000 males yearly [3]. There are many subtypes of priapism, with the most common one being an ischaemic subtype (95%) that is marked by pain and corporal rigidity. Ischaemic or low-flow priapism is viewed as an emergency due to tissue hypoxia and resultant acidosis, which can lead to corporal damage and resultant complete ED [1]. On histopathological examination, interstitial oedema and endothelial disruption occurs at 12 h after the onset of priapism; at 48 h, pathological progression to thrombi and smooth muscle necrosis occurs [4].

For treatment of ischaemic priapism, aspiration of dark corporal venous blood must be performed, with or without corporal irrigation with saline, until bright red blood is aspirated and visualised [5]. This aspiration has a modest complete success rate, not exceeding 30% [6]. Next in line for treating ischaemic priapism is corporal injection of sympathomimetic drugs, with the first option being phenylephrine due to its low cardiac side-effects secondary to minimal β-adrenergic side-effects, with preferable vital and/or cardiac rhythm monitoring [3,6,7]. Phenylephrine is given in aliquots of 200 µg every 5–10 min and not exceeding 1 mg every 1 h [8]. The authors are inclined to believe that cavernosal injections in patients with prolonged priapism will be unsuccessful if aspiration of the old blood/blood clots has not taken place, so that the medication can easily diffuse between the cells of the corpora.

Failure of resolution of priapism by sympathomimetic agents has no strict timeline, and there is no evidence about when to deem treatment with cavernosal injections pointless. It is suggested that prolonged treatment is futile due to smooth muscle paralysis from cellular damage [2]; however, in our present case we found that after every series of injections we got a partial response before painful erection returned, indicating that cellular damage had not occurred.

To the authors’ best knowledge, continuous irrigation with a sympathomimetic drug has not been reported in the literature before, and has shown to be successful in our present case. The authors understand the reluctance of physicians to administer continuous α-adrenergic drug due to the risk of necrosis; however, our dosage was the same as that reported in the literature. Continuous irrigation could at the molecular level saturate the α-adrenergic receptors rendering it more effective. Conceptually, there is an increased risk of systemic absorption of the sympathomimetic drug, which can cause tachyarrhythmias and elevated blood pressure, which were accounted for by continuous telemetry and vital signs monitoring [9]. Another risk of continuous infusion is regional penile ischaemia and local infection, which did not occur in this particular case, but were monitored for and did not happen. The authors used phenylephrine since AUA and European Association of Urology (EAU) guidelines propose phenylephrine as the first drug of choice for intermittent injections [3,4], whilst other agents could similarly be efficacious but they were not tried.

In conclusion, priapism is an emergency that is rarely encountered by general urologists. Adherence to management guidelines should be encouraged; however, it is still a disease entity that is not well understood and new treatment protocols may have a role in the future, including continuous intracavernosal adrenergic agent infusion as a last resort before surgical treatment. However, this is a pilot ischaemic priapism patient with positive results; further studies are needed to test its efficacy and safety profile.

## References

[1] Broderick GA, Kadioglu A, Bivalacqua TJ, et al Priapism: pathogenesis, epidemiology, and management. J Sex Med. 2010;7:476–500. 20092449 10.1111/j.1743-6109.2009.01625.x

[2] Ridgley J, Raison N, Sheikh MI, et al Ischaemic priapism: a clinical review. Turk J Urol. 2017;43:1–8. 28270944 10.5152/tud.2017.59458 PMC5330261

[3] Montague DK, Jarow J, Broderick GA, et al American Urological Association guideline on the management of priapism. J Urol. 2003;170:1318–1324. 14501756 10.1097/01.ju.0000087608.07371.ca

[4] Salonia A, Eardley I, Giuliano F, et al European Association of Urology. European Association of Urology guidelines on priapism. Eur Urol. 2014;65:480–489. 24314827 10.1016/j.eururo.2013.11.008

[5] Song PH, Moon KH. Priapism: current updates in clinical management. Korean J Urol. 2013;54:816–823. 24363861 10.4111/kju.2013.54.12.816 PMC3866283

[6] Huang YC, Harraz AM, Shindel AW, et al Evaluation and management of priapism: 2009 update. Nat Rev Urol. 2009;6:262–271. 19424174 10.1038/nrurol.2009.50 PMC3905796

[7] Pryor J, Akkus E, Alter G, et al Priapism. J Sex Med. 2004;1:116–120. 16422992 10.1111/j.1743-6109.2004.10117.x

[8] Kovac JR, Mak SK, Garcia MM, et al A pathophysiology-based approach to the management of early priapism. Asian J Androl. 2013;15:20–26. 23202699 10.1038/aja.2012.83 PMC3739130

[9] Muruve N, Hosking DH. Intracorporeal phenylephrine in the treatment of priapism. J Urol. 1996;155:141–143. 7490814

